# Strategy for Designing Selective Lysosomal Acid α-Glucosidase Inhibitors: Binding Orientation and Influence on Selectivity

**DOI:** 10.3390/molecules25122843

**Published:** 2020-06-19

**Authors:** Atsushi Kato, Izumi Nakagome, Mizuki Hata, Robert J. Nash, George W. J. Fleet, Yoshihiro Natori, Yuichi Yoshimura, Isao Adachi, Shuichi Hirono

**Affiliations:** 1 Department of Hospital Pharmacy, University of Toyama, Toyama 930-0194, Japan; s1560242@ems.u-toyama.ac.jp (M.H.); adachi@med.u-toyama.ac.jp (I.A.); 2 School of Pharmaceutical Sciences, Kitasato University, Tokyo 108-8641, Japan; nakagomei@pharm.kitasato-u.ac.jp (I.N.); hironos@pharm.kitasato-u.ac.jp (S.H.); 3 Institute of Biological, Environmental and Rural Sciences, Plas Gogerddan, Aberystwyth, Ceredigion SY23 3EB, UK; robert.nash@phytoquest.co.uk; 4 Chemistry Research Laboratory, Department of Chemistry, University of Oxford, Oxford OX1 3TA, UK; george.fleet@sjc.ox.ac.uk; 5 Faculty of Pharmaceutical Sciences, Tohoku Pharmaceutical University, Sendai 981-8558, Japan; natori-y@tohoku-mpu.ac.jp (Y.N.); yoshimura@tohoku-mpu.ac.jp (Y.Y.)

**Keywords:** iminosugars, glucosidase inhibitor, lysosomal acid α-glucosidase, ER α-glucosidase II, ligand docking, molecular dynamics, drug design

## Abstract

Deoxynojirimycin (DNJ) is the archetypal iminosugar, in which the configuration of the hydroxyl groups in the piperidine ring truly mimic those of d-glucopyranose; DNJ and derivatives have beneficial effects as therapeutic agents, such as anti-diabetic and antiviral agents, and pharmacological chaperones for genetic disorders, because they have been shown to inhibit α-glucosidases from various sources. However, attempts to design a better molecule based solely on structural similarity cannot produce selectivity between α-glucosidases that are localized in multiple organs and tissues, because the differences of each sugar-recognition site are very subtle. In this study, we provide the first example of a design strategy for selective lysosomal acid α-glucosidase (GAA) inhibitors focusing on the alkyl chain storage site. Our design of α-1-*C*-heptyl-1,4-dideoxy-1,4-imino-l-arabinitol (LAB) produced a potent inhibitor of the GAA, with an IC_50_ value of 0.44 µM. It displayed a remarkable selectivity toward GAA (selectivity index value of 168.2). A molecular dynamic simulation study revealed that the ligand-binding conformation stability gradually improved with increasing length of the α-1-*C*-alkyl chain. It is noteworthy that α-1-*C*-heptyl-LAB formed clearly different interactions from DNJ and had favored hydrophobic interactions with Trp481, Phe525, and Met519 at the alkyl chain storage pocket of GAA. Moreover, a molecular docking study revealed that endoplasmic reticulum (ER) α-glucosidase II does not have enough space to accommodate these alkyl chains. Therefore, the design strategy focusing on the shape and acceptability of long alkyl chain at each α-glucosidase may lead to the creation of more selective and practically useful inhibitors.

## 1. Introduction

Glycosidases are involved in a wide range of important biological processes; these include carbohydrate catabolism in the intestines, lysosomal catabolism of glycolipids, biosynthesis of oligosaccharide chains, and quality control of *N*-linked glycoproteins in the endoplasmic reticulum (ER). Accordingly, the inhibition of these glycosidases can have profound effects on the intestinal digestion, catabolism of glycolipids in lysosome, the maturation, transport, and secretion of glycoproteins, and can alter cell-cell or cell-virus recognition processes. This provides a basis for the potential use of glycosidase inhibitors in diabetes, genetic lysosomal disorders, cancer, and viral infection [1,2,3].

**Figure 1:**
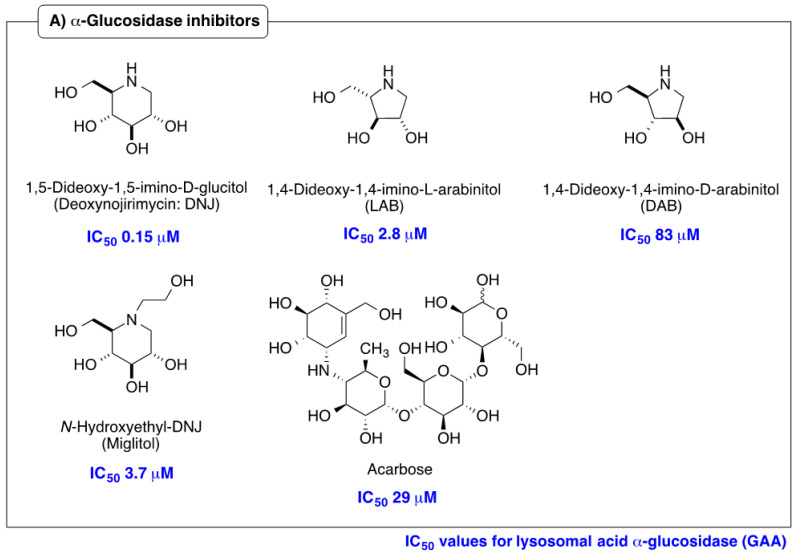

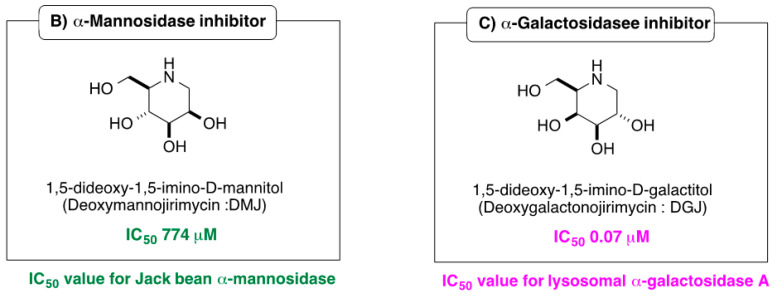
Structures and glycosidase inhibition of piperidine and pyrrolidine iminosugars, and acarbose. (**A**): α-glucosidase inhibitors. (**B**): α-mannosidase inhibitor. (**C**): α-galactosidase inhibitor.

Iminosugars are sugar mimics with a nitrogen atom in place of the ring oxygen of monosaccharides. They usually bind to the active sites of glycosidases in a reversible and competitive manner, as a result of their structural resemblance to the terminal sugar moiety in the natural substrates. A large number of iminosugars have been discovered from natural sources. They are classified into five structural classes: polyhydroxylated pyrrolidines, piperidines, indolizidines, pyrrolizidines and *nor*-tropanes [4,5]. Among them, the inhibition specificity of piperidine iminosugars (Figure 1) is usually easy to predict from the configurations of hydroxy groups, which are identical to those of the corresponding pyranose. For example, 1,5-dideoxy-1,5-imino-d-glucitol (deoxynojirimycin (DNJ)) corresponding to glucose in the pyranose configuration has high inhibition specificity against α-glucosidases. Similarly the polyhydroxylated piperidine analogues of mannose and galactose, 1,5-dideoxy-1,5-imino-d-mannitol (deoxymannojirimycin (DMJ)), and 1,5-dideoxy-1,5-imino-d-galactitol (deoxygalactonojirimycin (DGJ)), show the expected inhibition of α-mannosidase and α-galactosidase, respectively [6]. The structural basis of the inhibition of glycosidases by these compounds is clear, but this inhibition selectivity based on high similarity with the substrate may cause side effects. The anti-diabetes drug, miglitol, was designed from DNJ to suppress postprandial hyperglycemia, by inhibiting the hydrolysis of disaccharides such as maltose, isomaltase, and sucrose in the brush border membrane, and delaying the absorption of carbohydrates from the small intestine. However, miglitol is nearly completely absorbed from the intestinal tract [7]. Therefore, it inhibited not only intestinal α-glycosidases, but also the processing ER enzymes α-glucosidase I and II [8]. Therefore, closely mimicking iminosugars can be expected to give potent inhibition, but may cause selectivity problems between glycosidase isozyme species.

This study is focused on lysosomal acid α-glucosidase (GAA; EC 3.2.1.20). GAA is a retaining *exo*-glucosidase responsible for the cleavage of the α-glycosidic bond of glycogen to release glucose. Pompe disease is caused by mutations in the gene that encodes the GAA; these mutations result in reduced cellular enzyme activity, and the progressive accumulation of glycogen in the lysosomes of heart, skeletal muscles, and other tissues [9]. At present, more than 300 different point mutations have been identified within the GAA gene [10]. These GAA mutations result in a decrease of enzyme stability, and a subsequent decrease in the amount of enzyme activity in lysosome. Pharmacological chaperones, such as some reversible competitive inhibitors, appear to be able to act as a template that stabilizes the native folding state in the ER by occupying the active site of the mutant enzyme, thus allowing its maturation and trafficking to the lysosome [11,12]. It was reported that DNJ can act as a pharmacological chaperone, while also showing inhibition of ER α-glucosidase I and II, which might be linked to its side effects [13,14].

Thus, our strategy has been to design effective pharmacological chaperones for Pompe disease that affect the stability of GAA, which not only show a strong affinity for GAA, but also do not interfere with glycoprotein processing. We herein describe that the glucosidase inhibitor 1,4-dideoxy-1,4-imino-l-arabinitol (LAB) with α-1-*C*-heptyl chain is a more selective inhibitor of GAA than DNJ. We also investigated the effect of α-1-*C*-alkyl chain length on the selectivity for GAA vs. ER α-glucosidase II. An additional aim was to demonstrate the molecular docking properties of α-1-*C*-alkyl-LAB with GAA.

## 2. Results and Discussion

### 2.1. Structural Similarity of Active Site between GAA and ER α-Glucosidase II

To date, various DNJ derivatives and stereoisomers have been synthesized and investigated for the inhibition activities toward GAA [15,16], and ER α-glucosidase II [17]; however, there are no reports of the selective inhibition of each enzyme. 

**Figure 2:**
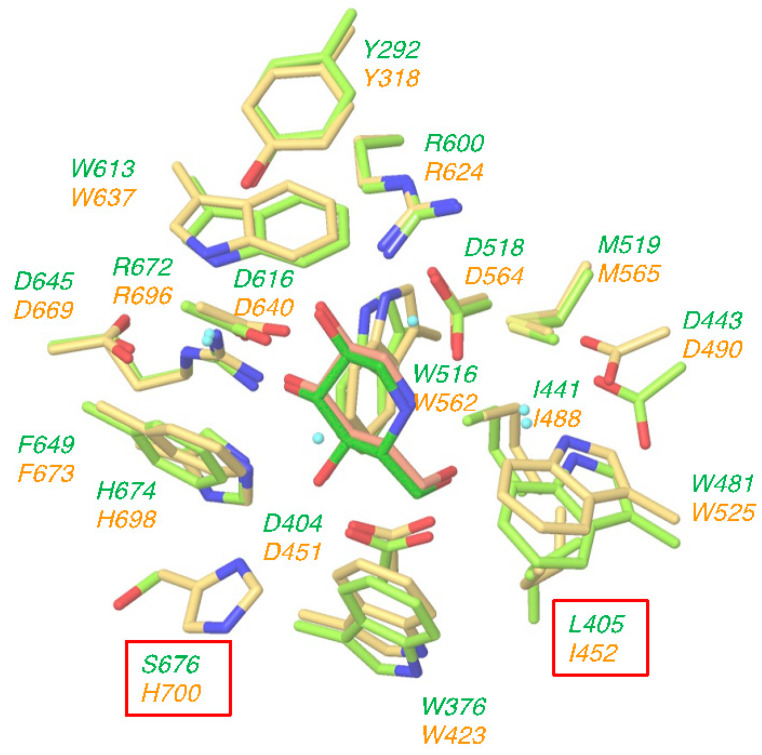
Structural overlap of lysosomal acid α-glucosidase (GAA) (light green) and endoplasmic reticulum (ER) α-glucosidase II (yellow) at the deoxynojirimycin (DNJ) binding site. Upper light green: amino acid residues of GAA. Lower yellow: amino acid residues of ER α-glucosidase II. Amino acid in a box: uncommon amino acid residues.

Several crystal structures of human GAA and mouse ER α-glucosidase II have been deposited in the Protein Data Bank. Among them, we selected the complex crystal structure of GAA with DNJ (PDB ID: 5NN5) and the complex crystal structure of ER α-glucosidase II with DNJ (PDB ID: 5IEE); these structures were superimposed on each other by using the Protein Structure Alignment tool in Maestro. Our selected complex crystal structures used DNJ as a common ligand, suitable for clarifying the different interactions between both enzymes. The amino acids around 5Å of DNJ are shown in Figure 2. The orientation and the conformation of DNJ are almost completely overlapped between GAA (light green) and ER α-glucosidase II (yellow). Moreover, as shown in Figure 2, amino acid residues surrounding the DNJ bound to the sugar recognition site of both enzymes are almost identical; the only two differences are that (i) Leu405 (GAA) has been replaced by Ile452 (ER α-glucosidase II) and (ii) Ser676 (GAA) has been replaced by His700 (ER α-glucosidase II). Furthermore, the hydrogen bonding, ionic and cation-π network between these enzymes and DNJ were very similar (see Supporting Information S1). These results suggested that the lack of specificity of DNJ may be attributed to the high degree of structural and sequence similarity of glucose recognition site of GAA with ER α-glucosidases. 

### 2.2. Influence of α-1-C-alkylation of LAB on Inhibition of GAA and ER α-Glucosidase II 

**Figure 3:**
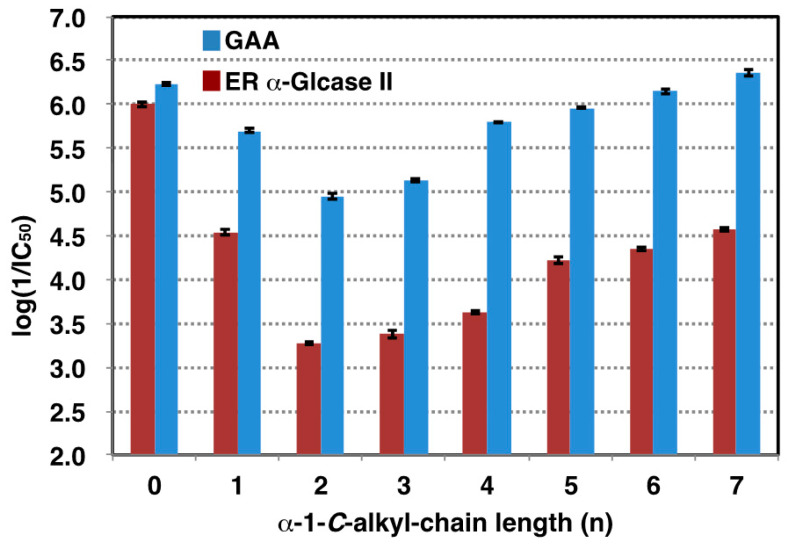
The effect of alkyl chain length on inhibitory activities against GAA (blue bar) and ER α-glucosidase II (red bar). Inhibition strength of GAA in each alkyl chain was indicated by log (1/IC_50_). n: α-1-*C*-alkyl-chain length.

On the basis of these findings, we turned our attention to the design of selective GAA inhibitors, with the goal of increasing the stability of GAA. Table 1 shows the 50% inhibitory concentrations (IC_50_) of LAB and its α-1-*C*-alkyl derivatives against GAA and ER α-glucosidase II. DNJ was used as a positive control. The parent compound, LAB, showed potent inhibitory activities against GAA and ER α-glucosidase II, with an IC_50_ value of 0.52 and 2.8 µM respectively. Inhibition potency of GAA was 3.5 times weaker than DNJ, whereas LAB was 2.9 times stronger inhibitor than GAA toward ER α-glucosidase II. Consequently, selectivity for GAA against ER α-glucosidase II of LAB was 10 times lower than DNJ. We next focused on the anomeric position and whether extending the alkyl chain at this position might cause differential inhibition by LAB. Figure 3 shows the logarithm of the reciprocal of pIC_50_ for α-1-*C*-alkyl-LAB against both enzymes as a bar graph. Of these compounds, with the introduction of a propyl instead of a methyl group, the inhibitory activity toward both enzymes tends to decrease, due to alkyl chain extension. Thereafter, both inhibitory activities were gradually recovered going from propyl to heptyl due to the elongation of the alkyl chain. However, it is notable that there is a large difference in the degree of reduction and recovery of the inhibitory activity for both enzymes. The effect of inhibition of GAA was small; even the most reduced α-1-*C*-ethyl-LAB was only 21-fold lower than LAB (Table 1). Furthermore, the inhibition of GAA was restored to the same level as LAB in α-1-*C*-heptyl-LAB. In contrast, the influence on ER glucosidase inhibition is large. α-1-*C*-Ethyl-LAB showed 193-fold weaker inhibition than LAB, and the inhibitory activity was not fully restored even when the alkyl chain was extended to heptyl. Consequently, α-1-*C*-heptyl-LAB displayed a remarkable selectivity index (SI) of 168.2, which is 31-fold better relative to the parent LAB (Table 1). 

### 2.3. Analysis of GAA- Ligand Stable Binding Pose by Using MD Calculation

**Figure 4:**
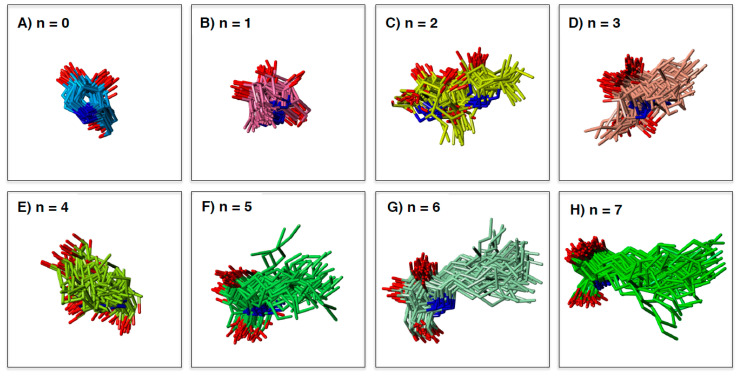
The binding poses of the LAB and α-1-*C*-alkyl-LAB derivatives extracted from the molecular dynamics (MD) trajectory: (**A**) LAB, (**B**) α-1-*C*-methyl-LAB, (**C**) α-1-*C*-ethyl-LAB, (**D**) α-1-*C*-propyl-LAB, (**E**) α-1-*C*-butyl-LAB, (**F**) α-1-*C*-pentyl-LAB, (**G**) α-1-*C*-hexyl-LAB, (**H**) α-1-*C*-heptyl-LAB.

**Figure 5:**
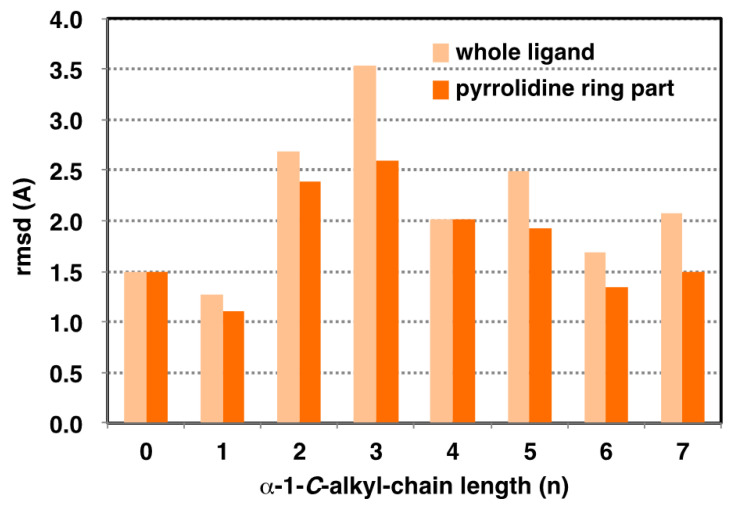
Effect of alkyl chain length on variation of ligand binding pose on GAA. Light orange: whole ligand (excluding hydrogen). Orange: pyrrolidine ring part (excluding hydrogen). Root mean square deviation (RMSD) value calculated for 50 extracted from 24 ns to 48 ns.

MD simulation can elucidate the dynamic changes in the interaction between target enzyme and the ligands. Maintaining a stable structure in the active site is an important factor for strong recognition by the enzyme. To understand the structural basis of the interaction of α-1-*C*-alkyl-LAB with GAA, we first studied the protein-ligand docking analyses of α-1-*C*-alkyl-LAB by using three GAA crystal structures (PDB ID: 5NN5, 5NN6, and 5NN8), which have reported as the complex structures of GAA with DNJ, miglitol, and acarbose, respectively. They commonly contain several water molecules in the ligand-binding site. Therefore, we considered these water molecules in the docking analyses. The docking analyses were performed using the grids generated from the crystal structures with two to four water molecule combinations in 5NN5 and 5NN6 and the crystal structure with two water molecules in 5NN8 (see Supporting Information S2). By including water molecules in the docking analyses, the docking poses of the co-crystalized ligands could be correctly reproduced. The best docking score poses were selected from many poses generated using each grid and energy-minimized to select one complex structure with the best binding interaction energy. Molecular dynamic (MD) simulations were carried out using the docking structure as initial structures. In the result of the MD simulation, the root mean square deviation (RMSD) of the protein heavy-atoms, compared with the initial structure, initially increased, and then became almost stable. The 50 ligand binding conformations, sampled equally in time intervals from the trajectories between 24 ns and 48 ns, were presented in Figure 4. Furthermore, Figure 5 shows the relationship between the extension of the α-1-*C*-alkyl chain length and the variation of the ligand binding pose by using the RMSD as the evaluation standard. We evaluated the variation of ligand binding pose from the viewpoint of the whole ligand (light orange) and the pyrrolidine ring part (orange). Among these compounds, the parent LAB and α-1-*C*-methyl-LAB remained the stable ligand-binding conformations, and their average RMSD values were both less than 1.5Å in the ligand-binding site (Figure 4A,B and Figure 5). In contrast, the introduction of short (ethyl and propyl) groups reduced the ligand-binding conformation stability and the positions of the pyrrolidine ring and the overall orientations moved drastically (Figure 4C,D). They resulted in the high variation of the position and the orientation of both the whole ligand and the pyrrolidine ring moiety (Figure 5). Furthermore, we found that the ligand-binding conformation stability gradually improved with increasing length of the alkyl chain; a much longer alkyl chain than butyl is required for achieving them (Figure 4E–H). Therefore, the introduction of longer (hexyl and heptyl) groups reduced the fluctuations of the ligand-binding conformation, and it was observed that the positions of the pyrrolidine ring were very stable (Figure 4G,H). 

### 2.4. Hydrogen Bonding Interactions and Cation-π Interactions between GAA and DNJ, LAB, and α-1-C-heptyl-LAB

**Figure 6:**
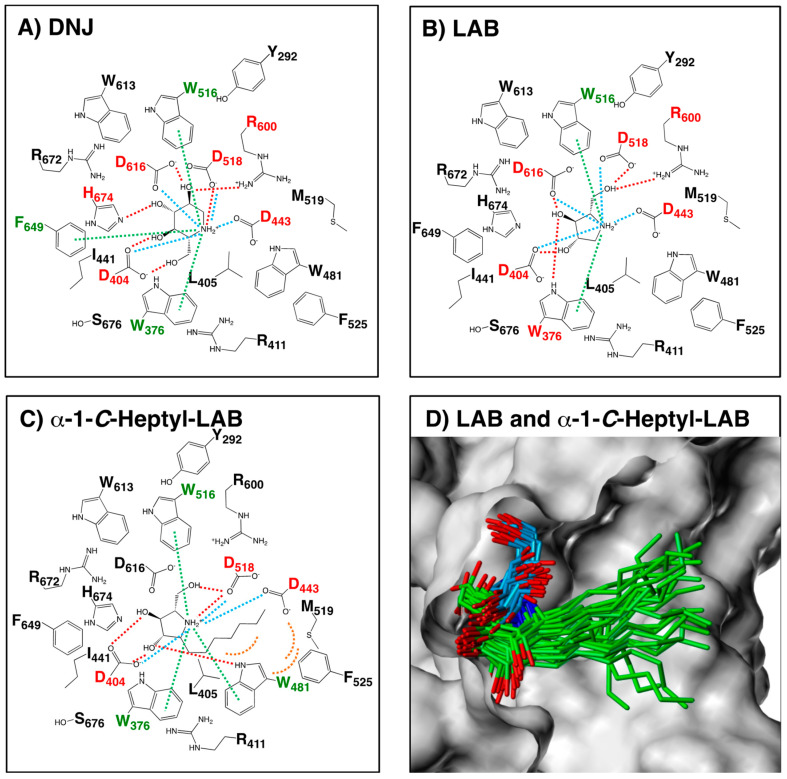
Schematic diagram of the interactions between GAA and (**A**) DNJ, (**B**) LAB and (**C**) α-1-*C*-heptyl-LAB. DNJ: the complex crystal structure (PDB ID: 5NN5). LAB and α-1-*C*-heptyl-LAB: the complex structure showing the best binding energy among 50 complex structures obtained from MD simulations. The red dashed lines represent hydrogen-bond interactions. The green dashed lines represent cation-π interactions. The blue dashed lines represent ionic interactions. The orange arcs (dashed line) represent hydrophobic interactions. The labels of amino acids involved in the hydrogen-bonding and the cation-π interactions are colored in red and green, respectively. (**D**) The 25 binding poses of LAB (blue) and α-1-*C*-heptyl-LAB (green) obtained from the MD simulations. The molecular surface of GAA is displayed using Sybyl-X 2.2.1 (Certara USA, Inc., Princeton, NJ, USA).

In order to investigate the interaction between GAA and the ligands, we modelled the three-dimensional structures of the complex structures by GAA with LAB and α-1-*C*-heptyl-LAB, using the docking protocol. As shown in Figure 6B, the positively charged nitrogen and the hydroxyl groups of LAB formed hydrogen bonds with Asp404, Asp518, Arg600, and Asp616. Furthermore, the pyrrolidine ring part of LAB had the favorable cation-π interaction with Trp376 and Trp516 and ionic interaction with Asp404, Asp443, Asp518, and Asp616. The schematic diagram of hydrogen bonding interactions and cation-π interactions between GAA and LAB was similar to that of DNJ (Figure 6A), except for the cation-π interaction with Phe649 and ionic interaction with His674 of DNJ. In contrast, α-1-*C*-heptyl-LAB formed clearly different interactions with GAA (Figure 6C). It formed the additional cation-π interaction and hydrogen bonds with Trp481, whereas it lacked hydrogen bonds with Asp616 and Arg600 and ionic interaction with Asp616. Furthermore, it is notable that the heptyl group of α-1-*C*-heptyl-LAB formed hydrophobic interaction with Trp481, Phe525, and Met519 of the alkyl chain storage pocket of GAA. Figure 6D showed the 25 binding poses of LAB (blue) and α-1-*C*-heptyl-LAB (green) obtained from the MD simulations. Both compounds reduced the fluctuations of the ligand-binding conformation, and the positions of the pyrrolidine ring were very stable. Therefore, the number of hydrogen bonds formed by α-1-*C*-heptyl-LAB is smaller than that of DNJ and LAB, but it was thought to be strongly affected by hydrophobic interactions.

### 2.5. Comparison of GAA and ER α-Glucosidase II Focusing on LAB-alkyl Chain Storage Pocket

**Figure 7:**
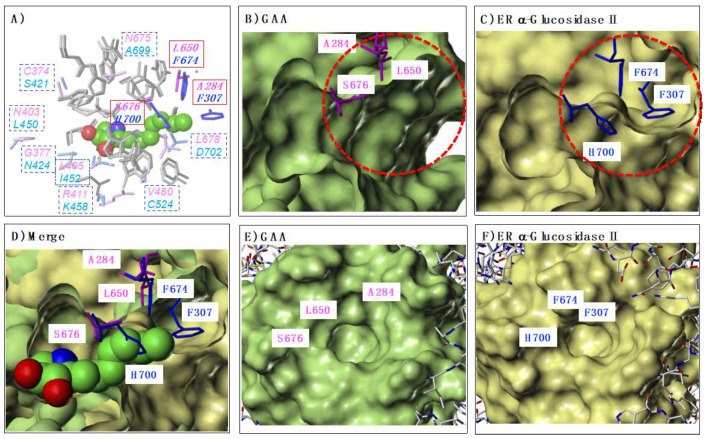
Difference of LAB-alkyl chain storage pocket in GAA and ER α-glucosidase II. (**A**) The amino acids of GAA and ER α-glucosidase II within 5Å surrounding the α-1-*C*-hexyl-LAB. The amino acids that differ between GAA (magenta) and ER α-glucosidase II (blue) were displayed in the square box. (**B**) The protein surface of GAA and the characteristic three amino acids, which composed the opening part (red dotted circle) of the active site. (**C**) The protein surface of ER α-glucosidase II and the characteristic three amino acids which composed the opening part (red dotted circle) of the active site. (**D**) The superimposition of each protein surface of GAA and ER α-glucosidase II. Docking pose of α-1-*C*-hexyl-LAB against GAA was superimposed with them. (**E**) GAA protein surface from the view of the outside of the opening part of the active site. (**F**) ER α-glucosidase II protein surface from the view of the outside of the opening part of the active site.

On the basis of these findings, we hypothesized that the difference in the storage sites of the alkyl chains would also affect the selectivity of GAA and ER α-glucosidase II. Based on this hypothesis, we first identified each storage site (Figure 7). At first, the crystal structures of GAA (PDB ID: 5NN5) and ER α-glucosidase II (PDB ID: 5IEE) were superposed using the Protein Structure Alignment tool in Maestro. Subsequently, the complex structure of each enzyme with α-1-*C*-hexyl-LAB was extracted from the MD simulation and superimposed on them (Figure 7). Figure 7A showed the amino acids of GAA and ER α-glucosidase II within 5Å surrounding the α-1-*C*-hexyl-LAB. The amino acids that differ between GAA (magenta) and ER α-glucosidase II (blue) were displayed in the square box. As shown in Figure 2, the amino acid residues surrounding the sugar recognition site of both enzymes were almost common. In sharp contrast, it was revealed that the amino acid residues surrounding the α-1-*C*-hexyl-LAB binding part in each active site was clearly different. We focused especially on three amino acids: Ala284, Leu650, and Ser676 (red solid square box), which composed the LAB-alkyl chain storage pocket. In case of ER α-glucosidase II, these amino acids were replaced by Phe307, Phe674, and His700. As shown in Figure 7B,C, the substitution of these three amino acids causes a large difference in the opening part (red dotted circle) of each active site. Similarly, from the view of the outside of the opening, it can be seen that GAA has a wider opening than ER α-glucosidase II (Figure 7E,F). Figure 7D showed the superimposition of both protein surface. Consequently, the space of the alkyl chain storage pocket in ER α-glucosidase II was narrower than GAA, and it cannot accommodate the extension of alkyl chains. 

### 2.6. GAA Stabilization Effect of LAB, α-1-C-ethyl-LAB and α-1-C-heptyl-LAB 

**Figure 8:**
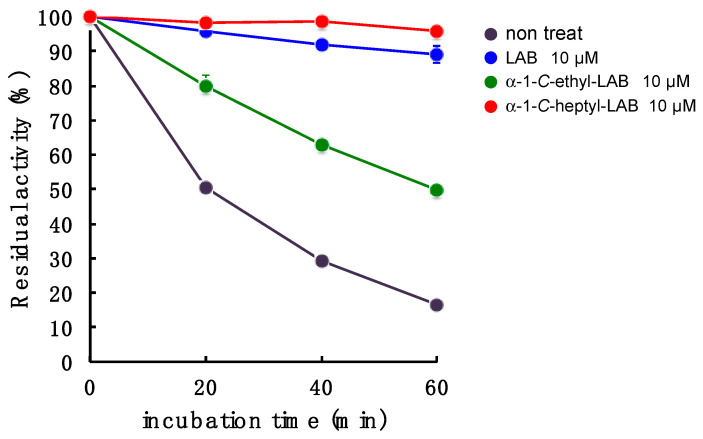
Effect of LAB, α-1-*C*-ethyl-LAB, and α-1-*C*-heptyl-LAB on the in vitro thermostability. GAA was incubated at 47 °C in 100 mM sodium citrate buffer (pH 6.8) containing 1 mg/mL of bovine serum albumin (BSA) at various incubation times with or without iminosugars. Each value represents the mean ± SEM (*n* = 3).

With respect to LAB and α-1-*C*-heptyl-LAB having a different binding pocket in the sugar-recognition site toward GAA, we next investigated the effect of these compounds on the stabilization of this enzyme at 47 °C (Figure 8). GAA was incubated in 100 mM McIlvaine buffer (pH 6.8) containing 0.25% sodium taurocholate and 0.1% Triton X-100 for 0, 20, 40, and 60 min, and the remaining enzyme activity was determined with 4-methylumbelliferl-α-d-glucopyranoside as substrate. The enzyme activity was 83% lost within 60 min under incubation without test samples, while the incubation with α-1-*C*-heptyl-LAB effectively stabilized GAA. The enzyme activity remained over 94% even under the incubation for 60 min in the presence of 10 µM α-1-*C*-heptyl-LAB (Figure 8). This behavior is similar to that of 10 µM LAB, but the stabilization effect was slightly weaker than LAB (89% activity remained at 60 min). Therefore, it is considered that α-1-*C*-heptyl-LAB forms an appropriate hydrogen ion network in the sugar-recognition site, which is different from LAB and DNJ, and stabilizes GAA. In contrast, 10 µM α-1-*C*-ethyl-LAB could not sufficiently protect the enzyme activity (50% activity remained at 60 min). These results correlate with stability in the MD analysis. 

## 3. Experimental Section

### 3.1. Chemistry

1,4-Dideoxy-1,4-imino-l-arabinitol (LAB) was prepared from l-arabinose, by an identical method to that used for the synthesis of 1,4-dideoxy-1,4-imino-d-arabinitol (DAB) [18,19,20,21]. α-1-*C*-Alkyl-LAB with various lengths of side chains from C1 to C7 at the 1-position were synthesized, as previously described [8]. 

### 3.2. Enzyme Inhibition Assays 

The inhibitory activity toward lysosomal acid α-glucosidase (GAA) was measured with Myozyme (Genzyme; Boston, MA, USA) as the enzyme source, and 4-methylumbelliferyl-α-d-glucopyranoside (Sigma-Aldrich Co.; St. Louis, Mo, USA) as substrate. The reaction mixture consisted 100 mM McIlvaine buffer (pH 6.8), 0.25% sodium taurocholate and 0.1% Triton X-100 (Nacalai Tesque Inc.; Kyoto, Japan), and the appropriate amount of enzyme. The reaction mixture was pre-incubated at 0 °C for 45 min, and the reaction was started by the using 4 mM substrate solution, followed by incubation at 37 °C for 30 min. The reaction was stopped by the addition of 1.6 mL of the solution of 400 mM Glycine-NaOH solution (pH 10.6). The released 4-methylumbelliferone was measured (excitation 362 nm, emission 450 nm) with a F-4500 fluorescence spectrophotometer (Hitachi, Tokyo, Japan). ER α-glucosidase II was prepared from the rat liver according to the method of Hino et al. [22]. For ER α-glucosidase II activities, 4-nitrophenyl-α-d-glucopyranoside (Sigma-Aldrich Co.; St. Louis, MO, USA) was used as substrate. The reaction mixture contained phosphate buffer (pH 6.8), 0.25% sodium taurocholate, and the appropriate amount of enzyme. The reaction mixture was pre-incubated at 0 °C for 30 min, and the reaction was started by the using 4 mM substrate solution, followed by incubation at 37 °C for 30 min. The reaction was stopped by adding 0.4 mL of 400 mM Na_2_CO_3_. The released p-nitrophenol was measured spectrometrically at 400 nm.

### 3.3. MD Calculations

The crystal structures of GAA with deoxynojirimycin, miglitol, and acarbose (PDB ID: 5NN5, 5NN6 and 5NN8) were downloaded from the Protein Data Bank. The preparations of the protein structures were performed using Maestro Protein Preparation Wizard (Schrödinger, LLC, New York, NY, USA). After removing water molecules other than those used in docking analysis, hydrogen atoms were added at pH 6.8. The docking analyses were performed with the standard precision (SP) mode of Glide (version 7.9, Schrödinger, LLC, New York, NY, USA) using the grids generated from the crystal structures with two to four water molecule combinations in 5NN5 and 5NN6 and the crystal structure with two water molecules in 5NN8. The best docking score poses were selected for each grid and energy-minimized to select one complex structure with the best binding interaction energy. These docking structures were solvated in a cubic simulation box using a simple point charge (SPC) water model with periodic boundary conditions. The net charge of the system was neutralized by addition of sodium and chloride ions. MD simulations were performed in 48 ns at 310 K and 1 bar and NPT ensemble using Desmond (version 5.4, Schrödinger, LLC, New York, NY, USA) [23,24]. Electrostatic interactions were calculated using the Particle Mesh Ewald with a cut-off distance of 9.0Å. MD simulations were performed using the OPLS3 force field. All calculations were performed using the Schrödinger Suite 2018-2 (Schrödinger, LLC, New York, NY, USA).

### 3.4. Thermostability of Lysosomal Acid α-Glucosidase (GAA) 

The enzyme GAA was incubated at 47 °C in 100 mM McIlvaine buffer (pH 6.8) containing 0.25% sodium taurocholate and 0.1% Triton X-100 for 0, 20, 40, and 60 min. After incubation, the remaining GAA activity was assayed immediately using 4 mM 4-methylumbelliferyl-α-d-glucopyranoside as substrate.

## 4. Conclusions

Iminosugars competitively inhibit glycosidases due to homology with the corresponding substrate. Therefore, piperidine iminosugars that faithfully mimic the stereochemistry of the terminal hexopyranose cleaved by *exo*-glucosidase are expected to show strong inhibition. However, it is difficult to define subtle differences in the isoenzyme that are localized in multiple organs and tissues. The present work elucidated the following features: (a) GAA and ER α-glucosidase II have a high degree of structural and sequence similarity at the glucose-recognition site; (b) LAB had the same level of inhibitory activity (IC_50_ = 0.52 µM) as DNJ (IC_50_ = 0.15 µM), and does not change recognition by GAA; (c) in contrast, the selectivity index (ER α-Glc II/GAA) of LAB was lower than DNJ, thus, the selectivity toward GAA cannot be obtained simply by changing the ring structure; (d) α-1-*C*-heptyl-LAB showed the most potent inhibition of GAA, with an IC_50_ value of 0.44 µM, furthermore, it displayed a remarkable selectivity (SI value of 168.2), which is 31-fold better than the parent LAB; (e) the MD simulation suggested that the ligand-binding conformation stability gradually improved with the increasing length of the alkyl chain, and a much longer alkyl chain than butyl is required for achieving this; (f) docking calculations suggested that the binding conformations and orientations of α-1-*C*-heptyl-LAB and monosaccharide mimics (DNJ and LAB) in GAA are clearly different—It had the lower number of hydrogen bonds, but it formed the characteristic hydrophobic interactions; (g) the amino acid residues surrounding the α-1-*C*-hexyl-chain storage part in each active site was clearly different—the space of ER α-glucosidase II was narrower than GAA, and it cannot accommodate the extension of alkyl chains; (h) α-1-*C*-heptyl-LAB improved the thermostability of GAA in vitro at the same level as LAB. 

Thus, our findings suggested that focusing on the alkyl chain storage site could improve the selectivity toward GAA and the ligand-binding conformation stability. 

## Figures and Tables

**Table 1:** IC_50_ values (µM) for α-1-*C*-alkyl-LAB and related compounds against processing ER α-glucosidase II (ER α-Glaase II) and lysosomal acid α-glucosidase (GAA).

			IC_50_ (μM)		Selectivity Index
	n =	Compounds	ER α-Glcase II	GAA	ER α-Glcase II/GAA
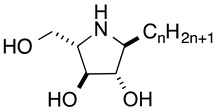	0	LAB	2.8 ± 0.22	0.52 ± 0.032	5.4
	1	α-1-*C*-Methyl-LAB	26 ± 2.0	2.0 ± 0.12	13.0
	2	α-1-*C*-Ethyl-LAB	541 ± 26	11 ± 1.1	49.2
	3	α-1-*C*-Propyl-LAB	413 ± 50	7.4 ± 0.31	55.8
	4	α-1-*C*-Butyl-LAB	228 ±13	1.6 ± 0.040	142.5
	5	α-1-*C*-Pentyl-LAB	180 ± 19	1.1 ± 0.088	163.6
	6	α-1-*C*-Hexyl-LAB	100 ± 6.2	0.71 ± 0.029	140.8
	7	α-1-*C*-Heptyl-LAB	74 ± 3.7	0.44 ± 0.044	168.2
		DNJ	8.0 ± 0.55	0.15 ± 0.0032	53.3

## Supplementary Materials

The following are available online, Figure S1: Interactions of deoxynojirimycin with GAA and ER α-glucosidase. Figure S2: Water molecules found in the ligand-binding site. The water molecules surrounded by dashed circles were used for the docking analyses.
