# Supplementary material for: Strategy for Designing Selective Lysosomal Acid α-Glucosidase Inhibitors: Binding Orientation and Influence on Selectivity
Source: Molecules. 2020 Jun 19;25(12):2843. doi: 10.3390/molecules25122843 (PMC7357040; doi:10.3390/molecules25122843)
Supplement: Supplementary file 1 [file molecules-25-02843-s001.pdf]

# Supporting Information S1

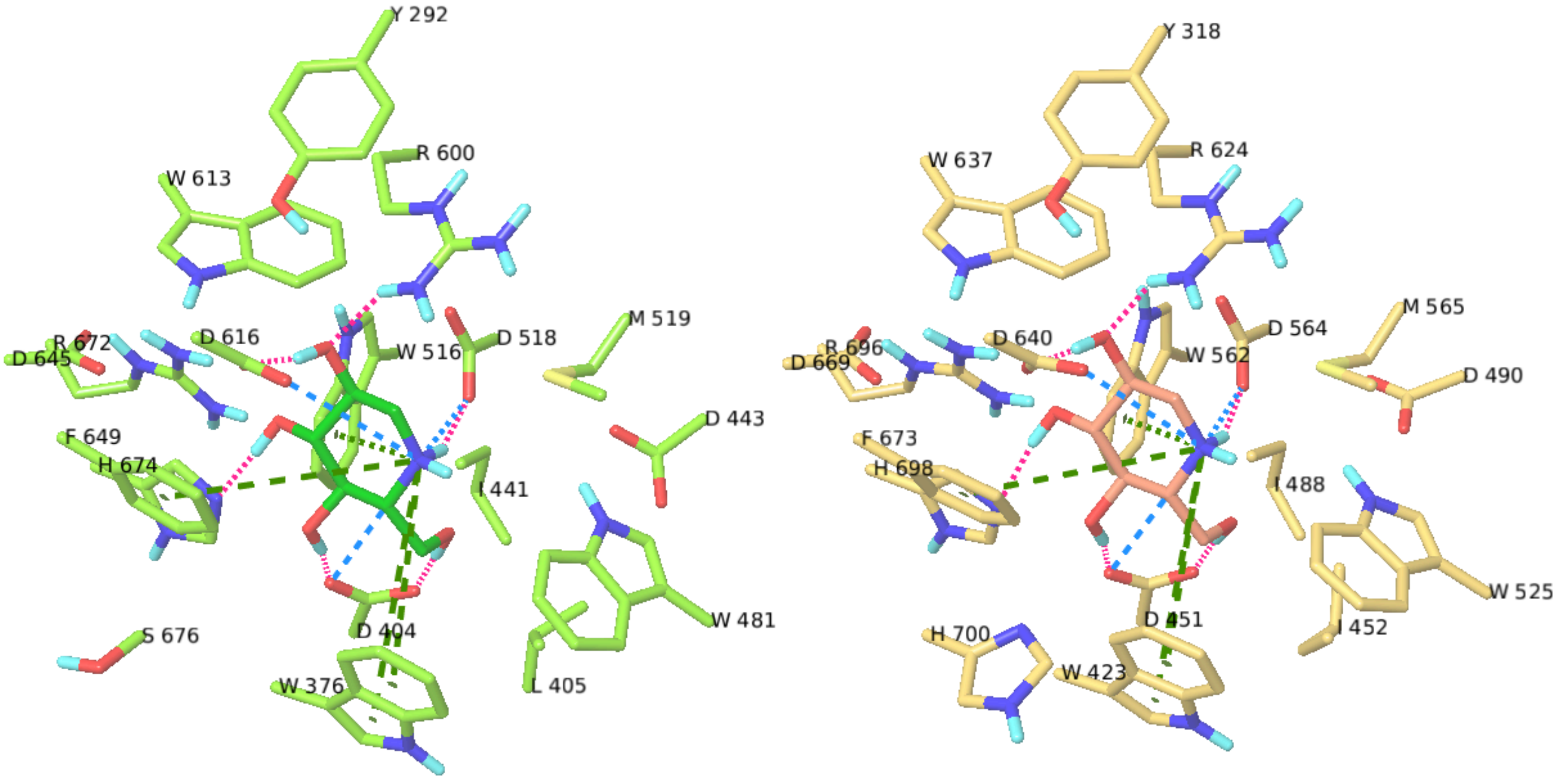


GAA (PDB ID: 5NN5)

ER α-glucosidases (PDB ID: 5IEE)

ligand :1-deoxynojirimycin
red dotted line: hydrogen bonds, blue dashed line: ionic, green dashed line: cation-π

Support Info
Interactions of 1-deoxynojirimycin with GAA and ER α-glucosidase.

Supporting Information S2

PDB ID: 5NN5 PDB ID: 5NN6 PDB ID: 5NN8

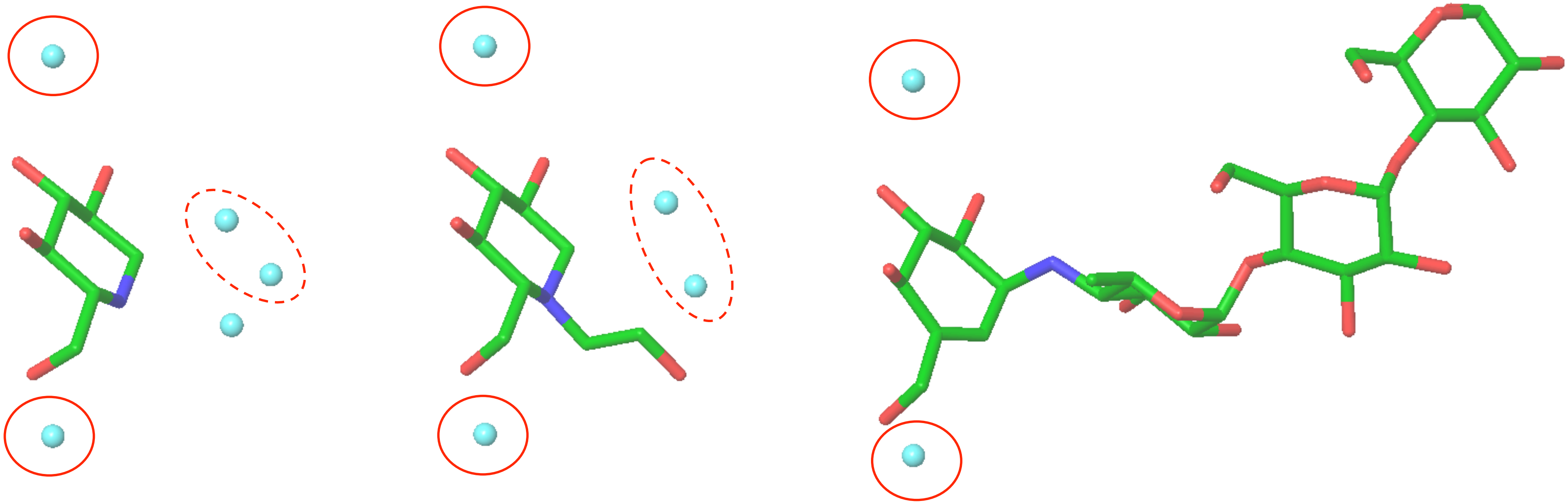

1-deoxynojirimycin miglitol acarbose

Support Info

Water molecules found in the ligand-binding site.

The water molecules surrounded by dashed circles were used for the docking analyses.
